# Inflammatory markers and clinical characteristics for predicting persistent positivity of interferon gamma release assay in dialysis population

**DOI:** 10.1038/srep34577

**Published:** 2016-10-05

**Authors:** Chin-Chung Shu, Chia-Lin Hsu, Chih-Yuan Lee, Vin-Cent Wu, Feng-Jung Yang, Jann-Yuan Wang, Chong-Jen Yu, Li-Na Lee

**Affiliations:** 1 Graduate Institute of Clinical Medicine, College of Medicine, National Taiwan University, Taipei, Taiwan; 2 Department of Traumatology, National Taiwan University Hospital, Taipei city, Taiwan; 3 Department of Internal Medicine, National Taiwan University Hospital, Taipei city, Taiwan; 4 Department of Surgery, National Taiwan University Hospital, Taipei City, Taiwan; 5 Department of Internal Medicine, National Taiwan University Hospital, Yun-Lin branch, Yun-Lin county, Taiwan; 6 Department of Laboratory Medicine, National Taiwan University Hospital, Taipei city, Taiwan

## Abstract

The interferon-gamma release assay (IGRA) is useful for diagnosing latent tuberculosis infection (LTBI), however the rate of negative conversion is high, especially in dialysis patients. Few studies have focused on predicting persistently positive patients who are at high risk of tuberculosis reactivation. We screened dialysis patients, and used QuantiFERON-TB Gold In-tube (QFT-GIT) to identify LTBI. Of the 157 participants who had initially positive QFT-GIT, 82 had persistently positivity and 75 had negative conversion. The persistently positive group were younger, more were current smokers, and had higher plasma level of soluble triggering receptor expressed on myeloid cells-1 (sTREM-1) and QFT-GIT responses than the negative conversion group. Multivariate logistic regression for persistent positivity revealed that high plasma sTREM-1 and QFT-GIT response, young age and TB contact history were independent factors. Currently smoking had borderline significance. The area under the receiver operating characteristic curve using the multi-factor model was 0.878, higher than 0.821 by QFT-GIT response of 0.95 IU/ml. In conclusion, dialysis patients with persistent LTBI status may be associated with a young age, high plasma sTREM-1, strong QFT-GIT response, currently smoking, and TB contact history. If resources are limited, these five predictors can be used to prioritize QFT-GIT-positive dialysis patients for LTBI treatment.

Tuberculosis (TB) remains one of the most important infectious diseases worldwide. According to World Health Organization (WHO) estimates, there were 9.6 million new TB cases and 1.5 million related deaths in 2014^1^. Control strategies include early treatment to prevent transmission and treatment of latent TB infection (LTBI) to reduce its reactivation^2^. Patients with renal failure undergoing dialysis are at an increased risk of TB due to attenuated cellular immunity^3^,^4^, and it has been reported that the risk of developing active TB is 7.8–25 times higher^5^,^6^,^7^ in dialysis patients compared to the general population. However, the diagnosis of TB is usually delayed because of frequent extra-pulmonary manifestations^8^,^9^. Thus, early LTBI detection in this specific group is important^2^.

Currently, interferon-gamma release assays (IGRAs) are used to diagnose LTBI. However, positive results are not 100% accurate for LTBI, and problems with variations in results have been reported^10^, IGRAs have several advantages^11^,^12^,^13^, including their application in immuno-compromised patients^14^, patients who have received the Bacillus Calmette–Guérin (BCG) vaccine^15^, and in areas where NTM is highly prevalent^16^. The IGRA has been shown to have a positive rate of around 21–40% in patients undergoing hemodialysis^17^,^18^,^19^,^20^. However, recent reports have shown a high negative conversion rate with the quantiFERRON Gold In-tube (QFT-GIT) test, a kind of IGRA, in cohorts of health care workers (33% after 18 weeks)^21^, subjects with close TB contact (35% after 6 months)^22^, and in patients undergoing dialysis (46% after 6 months)^10^.

Although inter-experiment variations have been reported to account for 8% of cases of negative conversion^23^, many other dynamic changes are also involved. Even healthy subjects with close TB contacts have been reported to have a 35% six-month negative conversion rate without preventive treatment^22^. These findings question the clinical significance of a single positive IGRA result, especially in patients undergoing dialysis. Many reports have suggested increasing the threshold of the QFT-GIT to avoid discordant results in the range of uncertainty^10^,^24^. On the other hand, follow-up IGRA testing has also been reported to be useful, and persistently positive IGRA results are more convincing^25^.

However, performing serial IGRAs has several disadvantages including the time required for follow-up, increased cost, and uncertainty until the results of the second test. Thus, identifying predictors of persistently positive IGRA results at the outset is important to classify those at high risk. Even though increasing the initial QFT-GIT threshold has been reported to predict persistent positivity, the sensitivity is only about 79%^10^. Thus, the aim of this study was to analyze clinical characteristics and serum cytokines from patients on dialysis with initial positive QFT-GIT test results to establish a model for predicting persistently positive LTBI results.

## Methods

We conducted this cohort study at National Taiwan University Hospital, a tertiary referral center, and its branches, regional teaching hospitals, and a local hemodialysis clinic. All of the study sites were located in northern Taiwan except for one in southern Taiwan. The Institutional Review Board of National Taiwan University Hospital approved the study. The study was conducted in accordance with approved guidelines, and all of the participants provided written informed consent.

Between May 2011 and April 2016, adult patients (age ≥20 years) with renal failure and treated with long-term (>3 months) dialysis were prospectively identified. Those with human immunodeficiency virus infection, liver cirrhosis of Child-Pugh class C^26^, cancer or autoimmune disease receiving chemotherapy within the last 3 months, and active TB within the last 3 years were excluded.

Peripheral blood was collected from the 981 participants at baseline. LTBI status was determined using a QuantiFERON-TB Gold In-tube assay (QFT-GIT) (Celestis, Australia) according to the manufacturer’s instructions^27^. We used a three-tube QFT-GIT kit, and QFT-GIT responses were calculated by subtracting the level of interferon-γ in the reaction supernatants of the negative control tube from that in TB-antigen tube. The results were defined as positive, negative, or indeterminate^28^,^29^.

**Figure 1:**
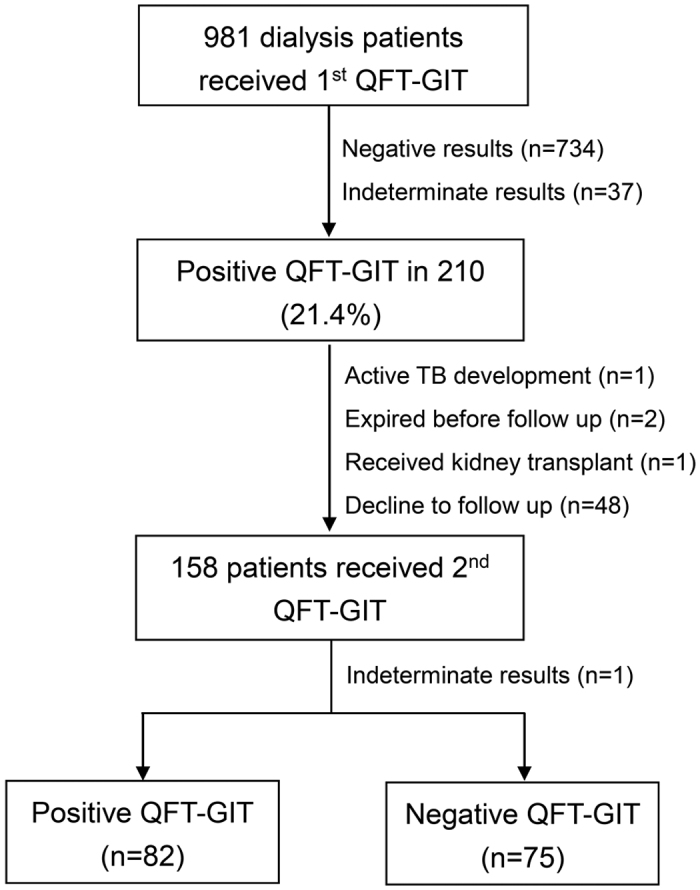
The flow chart of participant enrollment.

According to the initial QFT-GIT test, 210 (21.4%) of the 981 enrolled cases had positive results. Among them, 158 cases had follow up of QFT-GIT, including 82 persistent positive results, 75 negative conversions and one becoming indeterminate (Fig. 1). We measured levels of cytokines in all plasma samples from all of the participants except for 24 in whom the markers were measured in the supernatant of the QFT-GIT NIL tube due to a small amount of collected plasma. Inflammatory markers including interferon-gamma (IFN-γ) (R&D Systems, Inc., MN, USA) and soluble triggering receptor expressed on myeloid cells-1 (sTREM-1) (Aviscera Bioscience, Inc., CA, USA), and anti-inflammatory markers (interleukin-10 [IL-10] [R&D Systems, Inc., MN, USA], and decoy receptor 3 [DcR3] [R&D Systems Europe, Abingdon, UK]) were measured using enzyme-linked immunosorbent assays.

### Data Collection

The demographic and clinical data, including age, sex, underlying co-morbidities, prior TB history, respiratory and constitutional symptoms, smoking status, blood hemoglobin and serum albumin levels were recorded in a standardized case report form. Cough ≥3 weeks was defined as chronic cough. Current smokers were defined as those who had smoked >100 cigarettes, with the last time of smoking within 1 month prior to the study^30^. A history of TB household contact was defined as previously sharing the same living space with an active pulmonary TB patient.

The radiographic findings were classified into “no lung parenchymal lesions”; “lung lesions not compatible with TB”; “lung lesions compatible with prior TB”, or “lung lesions, cannot be excluded for TB”. “Lung lesions compatible with TB” was defined as new patches of consolidation, collapse, lymphadenopathy, mass or nodule, or cavitary lesion without any other proven etiology^7^. Prior TB was defined radiographically as fibrotic infiltrates with pleural thickening or calcified nodules over the upper lung fields, or other fibrotic lesions documented from previous TB^31^.

### Statistical Analysis

Inter-group differences were analyzed using the Student’s *t* test for numerical variables and the *chi*-square test for categorical variables. Multivariate logistic regression analysis was used to identify factors associated with persistently positive IGRA results. All potential predictors were included in backward conditional stepwise selection. A two-sided *p* < 0.05 was considered to be statistically significant. The discriminative power of each biomarker for persistently positive QFT-GIT was determined using receiver operating characteristic (ROC) curve and area under the curve (AUC) analyses. The optimal cut-off value, defined as the one with the least (1 − sensitivity)^2^ + (1 − specificity)^2^, was used to calculate sensitivity and specificity. All analyses were performed using SPSS software version 19.0 (SPSS, Chicago, IL).

## Results

Among the 157 dialysis patients with initial positive QFT-GIT, 82 had persistently positive QFT-GIT results and 75 had negative conversions. The average age of the persistently positive group was 60.2 years, which was lower than that in the negative conversion group (64.0 years, p = 0.032) (Table 1). The persistently positive group had a higher proportion of current smokers (27% vs. 13%, p = 0.036). Other clinical characteristics including sex, dialysis mode and duration, history of prior TB or TB contact, underlying diabetes mellitus, radiographic lesions, and respiratory symptoms were comparable between the two groups. With regards to the laboratory data (Table 2), blood hemoglobin was similar between the two groups, however albumin level was higher in the persistently positive group (4.1 vs. 4.0 g/dL, p = 0.042). Plasma level of the sTREM-1 was higher in the persistently positive group (594.8 vs. 183.1 pg/ml, p = 0.047), however there were no significant differences in anti-inflammatory markers (IL-10 and DcR3) and plasma IFN-γ between the two groups. In addition, the QFT-GIT response, calculated by subtracting the IFN-γ level in the negative control tube from the TB-antigen tube, was higher in the persistently positive group (3.8 vs. 1.1 IU/ml, p < 0.001).

In multivariate analysis for the predictors of persistently positive QFT-GIT results (Table 3), a higher level of serum sTREM-1 (OR: 1.001, 95% CI: 1.000–1.002, per 1 pg/ml increment), history of TB contact (OR: 5.040, 95% CI: 1.049–24.201, versus absence), age (OR: 0.939, 95% CI: 0.901–0.979, per year increment), and QFT-GIT response (OR: 2.450, 95% CI: 1.672–3.590, per 1 IU/ml increment) were significantly associated with persistent positivity. Although currently smoking (OR: 2.460, 95% CI: 0.896–6.759, versus ex- or non-smoking) was only borderline statistically significant, it was entered into the final multivariate model. The probability of persistently positive QFT-GIT results was calculated according to the model equation (1):


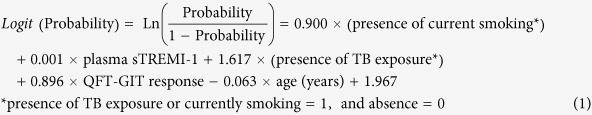


**Figure 2:**
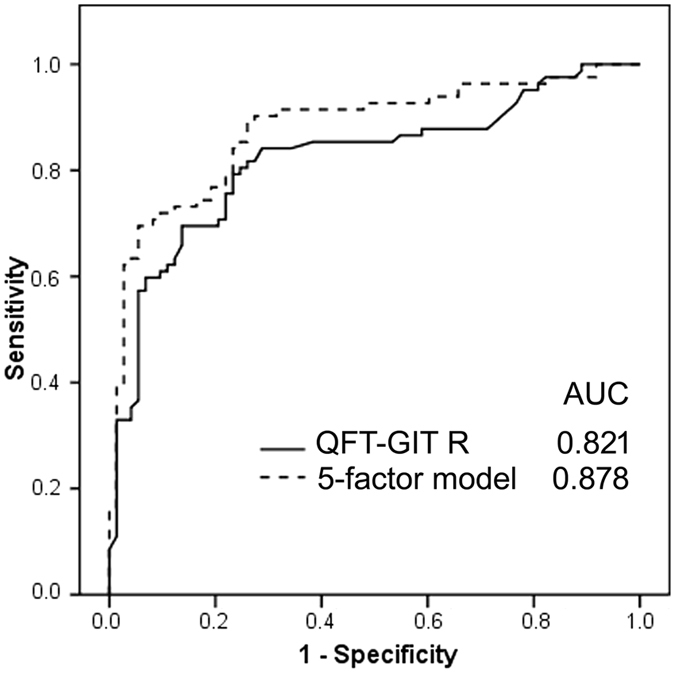
The receiver operating characteristic (ROC) curves according to different factors for predicting persistently positive quantiFERON-TB Gold In-tube results initially and 6 months later. AUC, area under the curve; QFT-GIT R, response of quantiFERON-TB Gold In-tube; 5-factor Model including serum sTREM-1, QFT-GIT R, history of TB contact, age and currently smoking.

Using QFT-GIT response and the 5-factor model to predict persistently positive IGRA results, we plotted receiver operating characteristic (ROC) curves (Fig. 2). The probability of the 5-factor model had an area under the curve (AUC) of 0.878, which was higher than that 1 by QFT-GIT response (0.821). The optimal values were 0.95 IU/ml and 0.42 for the initial QFT-GIT response and the probability of the 5-factor model, respectively. Cost and performance analyses are shown in Table 4. Using a higher QFT-GIT cut-off value of 0.95 IU/m could detect 79% of the persistently positive case in follow-up QFT-GIT. The 5-factor model could also improve the sensitivity and specificity with a smaller increase in cost (5.7 USD) than the QFT-GIT assay alone.

## Discussion

In the present study, we followed dialysis patients with an initially positive QFT-GIT result, and found a high negative conversion rate of 48% after 6 months. Compared to those with negative conversion of the QFT-GIT result, those with persistently positive results were younger and had a higher rate of current smokers as well as higher levels of albumin, plasma sTREM-1 and QFT-GIT responses than those with negative conversion. In multivariate analysis, QFT-GIT response, sTREM-1, age and history of TB exposure were independent risk factors for a persistently positive result. The multi-factor model could predict persistently positive IGRA results with a sensitivity of 84% and specificity of 77%.

The high negative conversion rate in this study is comparable to previous reports^10^,^21^,^22^, and deeply influences the application of the IGRA because the temporal positivity might be considered to exclude patients from preventive therapy. Due to the high cost and time of repeat IGRAs, some markers at the first assay have been suggested to predict persistent positivity. Among them, a high initial QFT-GIT response has been reported to be an indicator of a reduction in “gray-zone” variations^22^. However, the sensitivity was only 79% in the present study, similar to a previous report^10^. We also evaluated clinical factors and laboratory markers, and found that a younger age, history of TB contact, and plasma sTREM-1 level were significant predictors as well as QFT-GIT response.

The AUC of the QFT-GIT response was 0.821 in the present study, which is comparable to a previous study (AUC 0.815)^10^. In comparison, the 5-factor model had an AUC of 0.878. This means that measuring the level of sTREM-1, at a cost of 5.7 USD, and recording data on age, smoking status and history of TB contact in addition to the QFT-GIT assay could increase the predictive accuracy of persistent positivity by 5% sensitivity more than using QFT-GIT alone while maintaining a similar specificity (77%). The positive and negative predicted values reached 80%, and it could save 47.5 USD per case for a repeat IGRA 6 months later. With regards to selecting targeted LTBI population for preventive therapy, good negative likelihood ratio (0.22) by the 5-factor model could screen out transiently positive LTBI (negative conversion) and save cost of LTBI treatment.

The inflammatory marker sTREMI-1 was significantly associated with persistent positivity in QFT-GIT. This may be because a higher inflammatory status with LTBI may persist for longer. TREM-1 is expressed on neutrophils and macrophages and is up-regulated during bacterial infections. In addition, it can trigger and amplify inflammatory responses. In contrast, although the level of IFN-γ was relatively higher in the persistently positive group, it was not significant in uni- or multivariate analysis. This is consistent with a previous report which showed that sTREM-1 was more specific than IFN-γ for mycobacteria infections^32^. Anti-inflammatory cytokines such as IL-10 and immuno-modulator of DcR3 did not seem to be statistically significant, which may be due to a less significant role in inflammation in LTBI.

TB household contact is a factor for both LTBI and active TB. Although few studies have reported a correlation with LTBI persistence, we suggest that definite TB household contact may result in a longer duration of exposure than those without TB contact, and this may explain the correlation with LTBI persistence. Currently smoking has been proven to be an important factor for LTBI and active TB^20^,^30^. However, it was only borderline significant for LTBI persistence in this study, and further studies are necessary to clarify its role. Old age is also a known risk factor for LTBI, however it had an opposite effect on persistent LTBI in the present study. A possible explanation for the lower persistence of positive QFT-GIT responses in the older dialysis patients may be due to immune dysfunction, which leads to a shorter period of positive IGRA results. This is an important issue for the application of IGRAs in dialysis patients, because the average age of dialysis patients is usually older.

The present study has some limitations. First, the case number is small and firm conclusions require future large-scale validation. Second, external generalization is limited in dialysis patients, and the use of the prediction model for other immune-compromised population needs further investigation.

In conclusion, the negative conversion rate of positive IGRA results in dialysis patients was as high as 48% in this study. To save cost and avoid a long duration of follow-up, the focus should be on those with a young age, high QFT-GIT response, high level of plasma sTREM-1, currently smoking and those with a history of TB household contact, as they have a high probability of being persistently positive in QFT-GIT.

## Additional Information

**How to cite this article**: Shu, C.-C. *et al*. Inflammatory markers and clinical characteristics for predicting persistent positivity of interferon gamma release assay in dialysis population. *Sci. Rep.* **6**, 34577; doi: 10.1038/srep34577 (2016).

## Figures and Tables

**Table 1:** Baseline clinical characteristics.

	Persistently positive QFT-GIT (n = 82)	Negative conversion of QFT-GIT (n = 75)	*p* value
Age, year	60.2 (10.9)	64.0 (10.9)	0.032
Male sex	50 (61%)	39 (52%)	0.257
Current smoking	22 (27%)	10 (13%)	0.036
Dialysis mode, HD	73 (89%)	63 (84%)	0.356
Dialysis age, year	6.4 (5.4)	5.7 (5.5)	0.417
Diabetes mellitus	26 (32%)	23 (31%)	0.888
Kidney transplant	2 (2%)	0	0.173
Prior TB history	9 (11%)	5 (8%)	0.344
History of TB household contact	9 (11%)	3 (4%)	0.100
Radiological lesions*	10 (11%)	7 (9%)	0.670
Presence of symptoms^ǂ^	20 (24%)	13 (17%)	0.278

Abbreviations: HD, hemodialysis; QFT-GIT, quantiFERON-TB Gold In-tube; TB, tuberculosis.

Data are presented as number (%) or mean (standard deviation).

*Represents radiological lesions, compatible with prior TB or TB cannot be excluded.

^ǂ^Indicates chronic cough, dyspnea, fever, and other constitutional symptoms.

**Table 2:** Laboratory results based on the status of following quantiFERON-TB Gold In-tube (QFT-GIT).

	Persistently positive QFT-GIT (n = 82)	Negative conversion of QFT-GIT (n = 75)	*p* value
Hemoglobin, g/dL	10.7 (1.4)	10.8 (1.5)	0.654
Serum albumin, g/dL	4.1 (0.3)	4.0 (0.3)	0.042
DcR3, pg/ml	1622.7 (1182.8)	1406.6 (1399.9)	0.298
Interferon-gamma, pg/ml	117.2 (417.4)	68.7 (327.5)	0.424
sTREM-1, pg/ml	594.8 (1824.5)	183.1 (300.8)	0.047
Interleukin-10, pg/ml	18.5 (29.3)	32.0 (178.6)	0.504
QFT-GIT response, IU/ml	3.8 (3.3)	1.1 (1.6)	<0.001

Abbreviations: DcR3, decoy receptor 3; sTREM-1, soluble triggering receptor expressed on myeloid cells-1.

Data are presented as mean (standard deviation).

**Table 3:** Multivariate logistic regression analysis for predicting persistently positive quantiFERON-TB Gold In-tube (QFT-GIT) among patients with initially positive results.

Characteristics	Multivariate	
	*p* value	OR (95% C.I.)
Age, year	0.003	0.939 (0.901–0.979)
Sex, male vs. female	0.643	
Smoking, current vs. non-smoking	0.081	2.460 (0.896–6.759)
Diabetes mellitus, presence vs. absence	0.950	
Dialysis mode, PD vs. HD	0.251	
Prior TB history, presence vs. none	0.402	
History of TB contact, presence vs. none	0.043	5.040 (1.049–24.201)
Radiologic lesions*, presence vs. none	0.597	
Symptoms^ǂ^, presence vs. none	0.559	
Hemoglobin, g/dL	0.350	
Serum albumin, g/dL	0.505	
DcR3, pg/ml	0.153	
Interferon-gamma, pg/ml	0.875	
sTREM-1	0.050	1.001 (1.000^†^–1.002)
Interleukin-10, pg/ml	0.729	
QFT-GIT response, IU/ml	<0.001	2.450 (1.672–3.590)

Abbreviations: DcR3, decoy receptor 3; HD, hemodialysis; PD, peritoneal dialysis; sTREM-1, soluble triggering receptor expressed on myeloid cells-1; TB, tuberculosis.

*Represents radiological lesions, compatible with prior TB or TB cannot be excluded.

^ǂ^Indicates chronic cough, dyspnea, fever, and other constitutional symptoms.

^†^1.00000018.

**Table 4:** Cost and effectiveness of different predictive markers.

Markers	Cost* (USD)	Cut-off value	Sen.	Spe.	PPV	NPV	LR+	LR−
1^st^ QFT-GIT R	47.5	0.95 IU/ml	79%	76%	78%	77%	3.30 (2.18–5.02)	0.275 (0.18–0.42)
5-factor model^ǂ^	53.2	0.42	84%	77%	80%	80%	3.56 (2.32–5.46)	0.22 (0.14–0.36)

Abbreviations: QFT-GIT R, the response of quantiFERON-TB Gold In-tube; Sen, sensitivity; Spe, specificity; PPV, positive predictive value; NPV, negative predictive value.

*Including assay cost only.

^ǂ^Includes QFT-GIT response, plasma sTREM-1, history of TB contact, age and currently smoking.

USD is calculated at an exchange rate of 31.6 to NTD on March 16, 2015.
